# Knowledge of Symptoms of Acute Myocardial Infarction, Reaction to the Symptoms, and Ability to Perform Cardiopulmonary Resuscitation: Results From a Cross-sectional Survey in Four Regions in Germany

**DOI:** 10.3389/fcvm.2022.897263

**Published:** 2022-05-16

**Authors:** Nadja Kartschmit, Benedikt Birnbach, Saskia Hartwig, Rafael Mikolajczyk

**Affiliations:** Institute for Medical Epidemiology, Biometrics and Informatics, Interdisciplinary Center for Health Sciences, Medical School of the Martin-Luther-University Halle-Wittenberg, Halle (Saale), Germany

**Keywords:** epidemiology, acute myocardial infarction, knowledge about symptoms, cardiopulmonary resuscitation, help-seeking behavior, first responder reaction, awareness

## Abstract

**Background:**

Ischemic heart disease affects 126 million individuals globally which illustrates the importance of finding ways to decrease mortality and morbidity in case of an acute myocardial infarction (AMI). Since knowledge of symptoms, correct reaction to symptoms, and ability to perform cardiopulmonary resuscitation (CPR) decreases the time from symptoms-onset to reperfusion, which leads to lower AMI mortality, we aimed to examine those factors and identify predicting variables in regions with low and high AMI mortality rates.

**Methods:**

We conducted a cross-sectional online survey including 633 respondents from the general population in four federal states in Germany with low and high AMI mortality and morbidity rates. We used uni- and multivariable regressions to find health-related and sociodemographic factors associated with knowledge, reaction to symptoms, and skills in CPR.

**Results:**

Out of 11 symptoms, the mean of correctly attributed AMI symptoms was 7.3 (standard deviation 1.96). About 93% of respondents chose to call an ambulance when witnessing an AMI. However, when confronted with the description of a real-life situation, only 35 and 65% of the participants would call an ambulance in case of abdominal and chest pain, respectively. The predicting variables for higher knowledge were being female, knowing someone with heart disease, and being an ex-smoker compared to people who never smoked. Higher knowledge was associated with adequate reaction in the description of a real-life situation and ability to perform CPR. Prevalence ratio for being able to perform CPR was lower in females, older participants, and participants with low educational level. About 38% of participants state to know how to perform CPR. Our results indicate rather no difference regarding knowledge, reaction to AMI symptoms, and ability to perform CPR among different regions in Germany.

**Conclusions:**

Knowledge of symptoms and first responder reaction including skills in CPR is inadequate when confronted with the description of a real-life situation. Educational health campaigns should focus on conveying information close to real-life situations. Interventions for enhancing ability to perform CPR should be compulsory in regular intervals. Interestingly, we found no difference regarding the factors in regions with high and low AMI mortality rates in Germany.

## Introduction

Ischemic heart disease (IHD) affects around 126 million individuals globally (1,655 per 100,000), the global prevalence is rising, and it is the leading cause of death and disability worldwide, as well as in Germany (1, 2). One main manifestation of IHD is myocardial infarction (AMI). When witnessing an AMI, a swift and appropriate reaction is crucial to decrease the time to reperfusion and therefore improve prognosis (3–5). In Germany, mortality of AMI differs across federal states. The highest mortality rates are observed in Eastern and the lowest in Southern and Western Germany (6). These differences are partly, but not fully explained by differences in the prevalence of cardiovascular risk factors (7). It is hypothesized that differences could also be due to the number of people able to perform cardiopulmonary resuscitation (CPR), knowledge of AMI symptoms, and behavior during AMI (8). However, to date it is unknown whether these factors differ in regions with different mortality rates. Additionally, few studies have examined which health-related and sociodemographic factors are associated with knowledge, reaction to AMI symptoms, and ability to perform CPR.

Awareness of differences in knowledge and reaction to AMI symptoms, as well as ability to perform CPR in regions with different AMI mortality rates, could refine interventions to improve the response when witnessing an AMI (9). Additionally, knowledge of influencing factors of this response is essential. The aim of this study was to investigate associations of knowledge and reaction to AMI symptoms, and ability to perform CPR with health, health behavior, and sociodemographic factors taking into account regions with different AMI mortality rates in Germany.

## Materials and Methods

### Study Population

In December 2020 we re-contacted 35,835 people from a former survey (9,319 in Saxony-Anhalt with one of the highest AMI mortality rate, 9,182 in Baden-Wuerttemberg, 7,104 in North-Rhine Westphalia, and 10,230 in Schleswig-Holstein, among the federal states with the lowest AMI mortality rates) (6). Of these 35,835 invited, 857 persons conducted the cross-sectional online survey (2.4%).

### Outcomes

For evaluating participant's knowledge of AMI symptoms, we included 11 symptoms of which 10 were symptoms of AMI and one was not, and asked the participants whether these symptoms were AMI symptoms. We chose “sudden visual disturbances” as trap question, as it is not usually a symptom of AMI. We calculated a knowledge score for each participant by adding up the participant's correct answers about AMI symptoms, resulting in a score with a theoretical range from 0 to 11. We did not calculate the score in case of missing values. Our used question format was chosen as it has been used regularly in current literature as well as in Germany (10, 11). The included set of symptoms are based on the ten most frequently asked symptoms and trap symptom in the current literature (11).

We evaluated the participant's reaction when witnessing a potential AMI by describing the following everyday scenario and giving different options to choose from:

You are talking on the phone to a female person you are close with. She is 60 years old and had no severe diseases to date. She says that she
1) suddenly has chest pain or
2) has abdominal pressure since an hour, but it is getting worse now. She feels weak and wants to lie down and rest. You say goodbye and finish the conversation.

The symptoms (chest or abdominal pain) were randomly assigned to the participants. The following answer options were given:

a) Chest pain/abdominal pain is common. You let her rest and decide to ask her about her health the next time you talk to her.
b) Chest pain/abdominal pain could be related to the heart. Lying down relieves the strain on the heart. You decide to call her back in 2 h and ask about her well-being.
c) Chest pain/abdominal pain could be a sign of a severe disease. You call her back and convince her to call an ambulance.
d) Do not know.
e) Something else (with a free-text field for describing the reaction).

Additionally, the survey included two multiple-choice questions with free-text fields that asked participants what they would do if they saw someone lying on the floor in a department store and what they would do if they thought someone had an AMI. We analyzed these two questions descriptively and did not use them as outcomes in regression.

For examining the ability to perform CPR we asked the participants whether they know how to perform CPR and gave the following answer options:

a) Yes, I can perform it myself or instruct someone else to perform it.
b) Yes, theoretically, but I do not dare to perform it in a real-life situation.
c) No, I have learned it, but do not remember well how to perform it.
d) No, I never learned or knew how to perform it.

We included the following variables as possible predictors for the above-described outcomes: (1) Gender (self-classified as male, female, diverse), (2) age in years, (3) educational level, (4) federal state, (5) having a heart disease, (6) knowing someone with a heart disease, (7) self-reported high blood pressure, and (8) smoking status. Additionally, we included the knowledge score as predictor for reaction to AMI symptoms and ability to perform CPR. We chose the variables based on thorough literature review (8, 11).

### Statistical Analysis

Data of 633 participants were analyzed after excluding observations with missing values for any of the included variables. Since there was only one diverse person, we excluded this observation in all analyses that included gender as variable.

For analyzing the reaction to AMI symptoms, we dichotomized the answer options. We chose convincing the women to call an ambulance as appropriate and all other options as less appropriate. We conducted all analyses separately for the symptoms chest and abdominal pain.

For analyzing the ability to perform CPR, we also dichotomized the answer options. We chose the answer option to be able to perform CPR or instruct someone else to perform it as one category, since this answer option reflects if someone would perform or help to perform it in a real-life situation. The other answer options were included in another category.

For the knowledge score, we performed linear regressions. For the binary outcomes, we estimated Prevalence Ratios (PRs) using binomial distribution with log link function, since the outcomes were common (35.4–64.6% for the certain outcomes) (12).

We conducted uni- and multivariable regressions to identify factors possibly associated with the different outcomes. Since there was no evidence for serious multicollinearity, we included all variables in multivariable regression (variance inflation factor < 5 for all variables) (13–17). Since there is some evidence in previous studies that the effect for age could be non-linear, we tested non-linear associations between age and the outcomes using thin plate splines in generalized additive models. Since non-linear associations did not improve model fit (Akaike Information Criteria <10 for linear vs. non-linear terms), we included age as linear term in all models (18). We used R-Studio V.3.4.4 and SAS V. 9.4 for the analyses (19, 20).

## Results

Females, males, and the four federal states were equally represented in the sample. One person was diverse. The mean age was 49 years. About 56% of the participants held a university degree (Bachelor or higher). About 10% of the participants reported to have a heart disease and more than half of the sample indicated to know a person with heart disease. About 35% of the participants stated to have hypertension (currently or in the past), which is about the percentage of people with hypertension in the general middle-aged German population (21). About half of the sample stated to be current or former smokers (Table 1).

**Table 1:** Sociodemographic characteristics and reaction to other people's symptoms of myocardial infarction (*n* = 633).

**Gender**	
Female, *n* (%)^a^	322 (50.9)
**Age**	
Age, mean (SD)	49.2 (15.3)
Below 30 years old, *n* (%)	85 (13.4)
30–39 years old, *n* (%)	110 (17.4)
40–49 years old, *n* (%)	102 (16.1)
50–59 years old, *n* (%)	153 (24.1)
60–69 years old, *n* (%)	124 (19.6)
70 years and older, *n* (%)	59 (9.3)
**Education**	
Master, diploma, doctorate (PhD), *n* (%)	213 (33.6)
Bachelor or equivalent, *n* (%)	139 (22.0)
Vocational training, *n* (%)	245 (38.7)
No degree/still in training or studying, *n* (%)	36 (5.7)
**Federal state**	
Baden-Wuerttemberg	152 (24.0)
North Rhine-Westphalia	150 (23.7)
Saxony-Anhalt	171 (27.0)
Schleswig-Holstein	160 (25.3)
**Disease related factors and risk factors of myocardial infarction**	
Disease related to heart, *n* (%)	66 (10.4)
Knowing person with heart disease, *n* (%)	359 (56.7)
High blood pressure (currently/in the past), *n* (%)	220 (34.8)
Current smoker, *n* (%)	99 (15.6)
Former smoker, *n* (%)	204 (32.2)
Never smoked, *n* (%)	330 (52.1)

a *One person was diverse. SD, standard deviation*.

**Figure 1:**
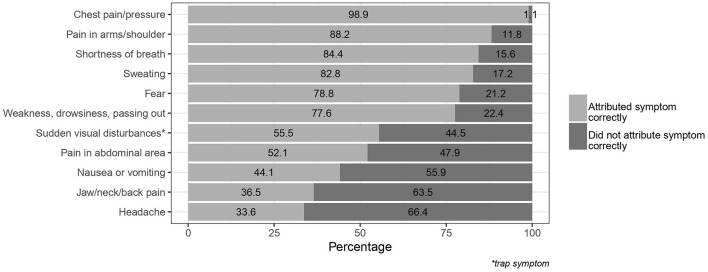
Symptoms of myocardial infarction identified as correct/incorrect by the participants; *trap symptom.

Almost all participants correctly identified chest pain/pressure as being a symptom of AMI. The four least known symptoms were abdominal pain, nausea/vomiting, headache, and jaw/neck/back pain (Figure 1; Supplementary Figure 1). In North Rhine-Westphalia, 63% correctly identified abdominal pain as symptom, while only 44% in Baden-Wuerttemberg did so (Supplementary Figure 1). The mean number of correctly attributed symptoms was 7.3 out of 11 symptoms (standard deviation 1.96).

Asking the participants what they would do when witnessing a person lying on the floor in a department store, about 10% would call an ambulance. About 40% stated to check whether the person was breathing before deciding to call an ambulance. Another 40% stated to start CPR, if necessary. About 6% would ask somebody else for help, as they did not feel capable of gauging the situation correctly. Almost all participants stated to call an ambulance when they assume that someone has an AMI (Supplementary Table 1).

**Figure 2:**
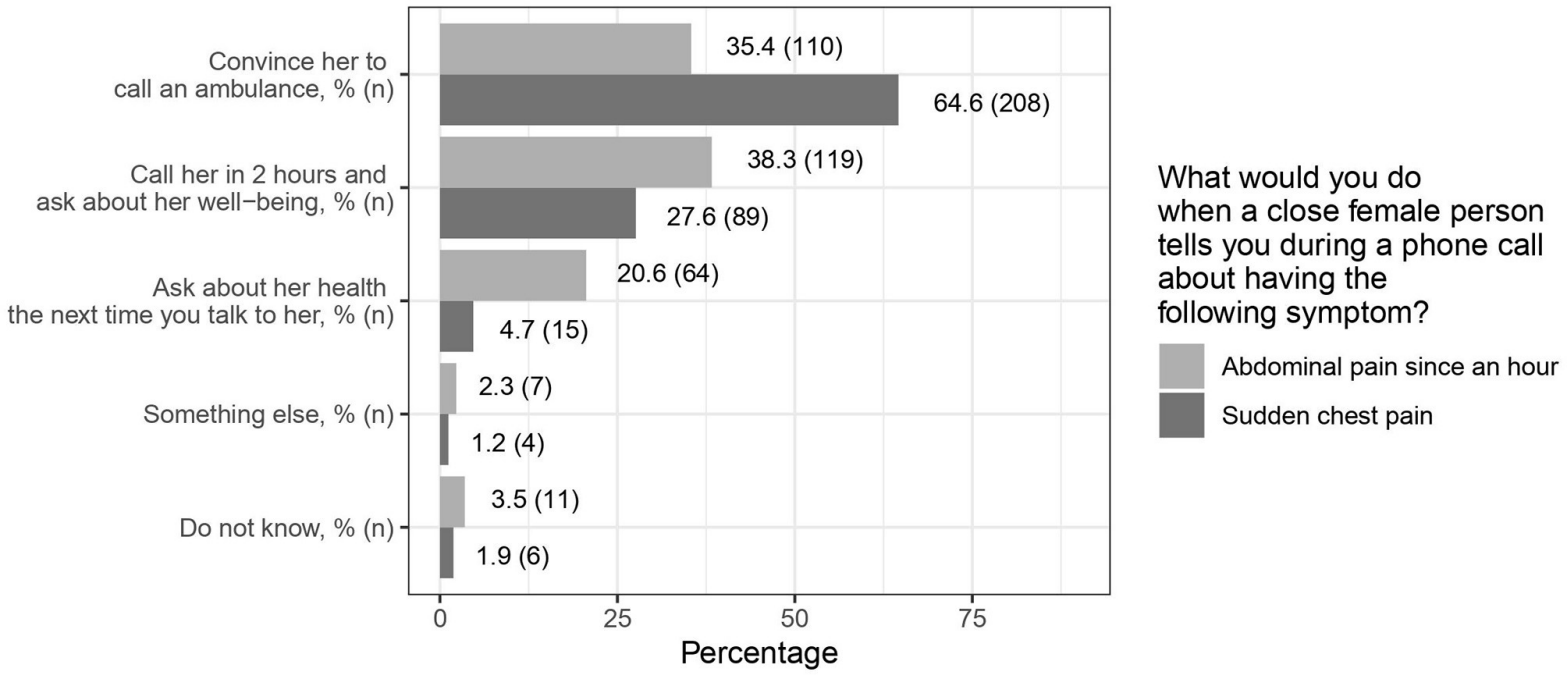
Reaction to two symptoms of myocardial infarction.

In the phone call scenario, 64% of the participants who received chest pain as symptom would convince the women to call an ambulance, while 35% of the participants who received abdominal pain would do so (Figure 2). This tendency was seen in all federal states. The proportion to convince the women to call an ambulance in both scenarios was highest in Saxony-Anhalt when compared to the other federal states (Supplementary Figure 2).

**Figure 3:**
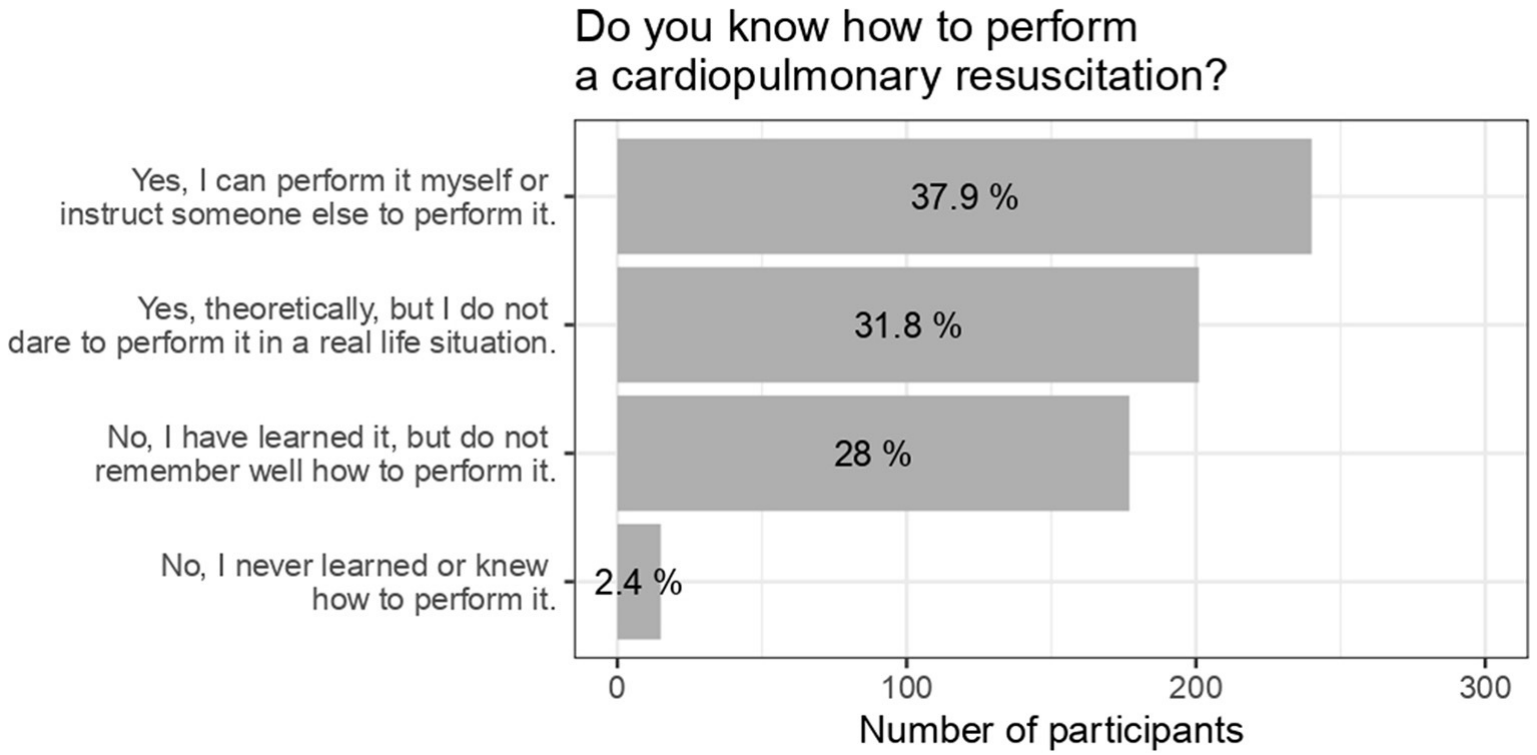
Ability to perform a cardiopulmonary resuscitation.

About 40% of the participants indicated that they could perform CPR themselves or instruct someone else to perform it. Around 30% indicated theoretically knowing how to perform it or have learned, but do not remember well how to perform it, respectively (Figure 3). The highest percentage regarding being able to perform CPR was in Schleswig-Holstein, while the highest percentage of theoretically knowing how to perform it was in Saxony-Anhalt (Supplementary Figure 3).

Examining the factors associated with knowledge of AMI symptoms, reaction to symptoms, and ability to perform resuscitation, we found that the mean knowledge score was higher in females when compared to males and lower in people aged 70 years or older when compared to the other age groups. The score was higher for people knowing someone with heart disease when compared to people not knowing someone with heart disease.

In the phone call scenario, the proportion of people choosing the adequate reaction in case of sudden chest pain was highest in the age groups 40–49 and 60–69 years when compared to the other age groups. The proportion was higher for people knowing someone with heart disease and in former smokers.

In case of abdominal pain, the proportion was highest in the age group 60–69 years and higher in people having heart disease.

Regarding the ability to perform CPR, more males than females reported to be able to perform it and the proportion decreased with increasing age. People with higher educational level reported more often to be able to perform CPR when compared to people with lower educational level. Higher proportion of former and current smokers reported to be able to perform CPR (Table 2).

**Table 2:** Knowledge score, reaction to other people's symptoms of myocardial infarction, ability to perform CPR and their possible predictors.

	**Knowledge score^b^**	**Would convince her to call ambulance in case of sudden chest pain (*n* = 322)^c^**	**Would convince her to call ambulance in case of abdominal pain (*n* = 311)^c^**	**Ability to perform CPR^d^**
	**Mean (SD)**	**% (*n*)**	**% (*n*)**	**% (*n*)**
Female^a^	7.71 (1.84)	64 (103/161)	36 (58/161)	36.0 (116/322)
Male^a^	6.93 (2.00)	65.6 (105/160)	34.7 (52/150)	40.0 (124/310)
Below 30 years old	7.42 (1.87)	57.5 (23/40)	35.6 (16/45)	44.7 (38/85)
30–39 years old	7.29 (2.00)	56.6 (30/53)	26.3 (15/57)	43.6 (48/110)
40–49 years old	7.52 (2.08)	71.7 (38/53)	26.5 (13/49)	39.2 (40/102)
50–59 years old	7.37 (1.89)	64.6 (51/79)	37.8 (28/74)	37.9 (58/153)
60–69 years old	7.33 (1.93)	72.9 (51/70)	48.1 (26/54)	30.6 (38/124)
70 years and older	6.78 (2.03)	55.6 (15/27)	37.5 (12/32)	30.5 (18/59)
Master, diploma, doctorate (PhD)	7.38 (1.92)	63.2 (67/106)	30.8 (33/107)	39.4 (84/213)
Bachelor or equivalent	7.27 (1.95)	61.8 (42/68)	42.3 (30/71)	41.7 (58/139)
Vocational training	7.28 (2.00)	67.7 (88/130)	32.2 (37/115)	35.9 (88/245)
No degree/still in training or studying	7.47 (1.99)	61.1 (11/18)	55.6 (10/18)	27.8 (10/36)
Baden-Wuerttemberg	7.13 (2.08)	58.6 (41/70)	26.8 (22/82)	35.5 (54/152)
North Rhine-Westphalia	7.69 (1.84)	69.1 (56/81)	30.4 (21/69)	38.7 (58/150)
Saxony-Anhalt	7.27 (1.96)	70 (63/90)	46.9 (38/81)	36.3 (62/171)
Schleswig-Holstein	7.22 (1.92)	59.3 (48/81)	36.7 (29/79)	41.3 (66/160)
Heart disease	7.36 (1.97)	67.6 (23/34)	40.6 (13/32)	34.8 (23/66)
Having no heart disease	7.32 (1.87)	64.2 (185/288)	34.8 (97/279)	38.3 (217/567)
Knowing someone with heart disease	7.61 (1.86)	67 (118/176)	35 (64/183)	39.8 (143/359)
Not knowing anyone with heart disease	6.94 (2.02)	61.6 (90/146)	35.9 (46/128)	35.4 (97/274)
High blood pressure	7.24 (1.89)	66.1 (78/118)	37.3 (38/102)	32.3 (71/220)
No high blood pressure	7.37 (2.00)	63.7 (130/204)	34.4 (72/209)	40.9 (169/393)
Never smoked	7.22 (2.00)	62.1 (105/169)	33.5 (54/161)	35.2 (116/330)
Former smoker	7.59 (1.88)	69.3 (70/101)	35.9 (37/103)	39.7 (81/204)
Current smoker	7.12 (2.00)	63.5 (33/52)	40.4 (19/47)	43.4 (43/99)
Knowledge score	–	7.4 (1.8)	8.1 (1.8)	8.0 (1.8)

a *Because of only 1 observation, the category “diverse” was not included in the analysis, n = 632*.

b *Higher score indicating higher knowledge, possible range: 0–11, minimum in the sample: 2, maximum in the sample: 11*.

c *Refers to question: What would you do when a close female person tells you during a phone call about having abdominal pain since an hour/sudden chest pain? The symptom was randomly assigned to the participants*.

d *Refers to question: Do you know how to perform a cardiopulmonary resuscitation? The answer option: “Yes, I can perform it myself or instruct someone else to perform it” was considered “ability to perform resuscitation.”*

*CPR, cardiopulmonary resuscitation; SD, standard deviation*.

### Results of Regression Analyses

Estimates for the certain factors were comparable in uni- and multivariable regressions (Table 3; Supplementary Table 2).

**Table 3:** Multivariable regressions for knowledge and reaction to symptoms of myocardial infarction and possible predictors (*n* = 632^a^).

	**Knowledge score, ß (95% CI)^b^**	**Would convince her to call ambulance in case of sudden chest pain (*n* = 322), PR (95% CI)^c^**	**Would convince her to call ambulance, in case of abdominal pain (*n* = 311), PR (95% CI)^c^**	**Ability to perform CPR, PR (95% CI)^d^**
Male^a^	Reference			
Female^a^	0.75 (0.44, 1.06)	0.93 (0.78, 1.10)	1.00 (0.73, 1.36)	0.77 (0.63, 0.94)
Age (per 10 years)	−0.08 (−0.19, 0.03)	1.03 (0.97, 1.10)	1.11 (1.00, 1.24)	0.91 (0.85, 0.98)
Master, diploma, doctorate (PhD)	Reference			
Bachelor or equivalent	−0.11 (−0.52, 0.30)	0.93 (0.72, 1.19)	1.26 (0.85, 1.85)	1.01 (0.79, 1.29)
Vocational training	−0.23 (−0.59, 0.13)	1.08 (0.89, 1.32)	0.93 (0.64, 1.35)	0.91 (0.70, 1.15)
No degree/still in training or studying	−0.15 (−0.85, 0.56)	0.94 (0.63, 1.40)	1.72 (0.96, 3.06)	0.56 (0.34, 0.93)
Saxony-Anhalt	Reference			
Baden-Wuerttemberg	−0.11 (−0.53, 0.31)	0.89 (0.70, 1.15)	0.66 (0.43, 1.01)	0.93 (0.71, 1.23)
North Rhine-Westphalia	0.29 (−0.13, 0.72)	1.00 (0.80, 1.26)	0.72 (0.48, 1.09)	0.93 (0.71, 1.22)
Schleswig-Holstein	−0.12 (−0.54, 0.29)	0.87 (0.68, 1.13)	0.81 (0.56, 1.19)	1.09 (0.84, 1.41)
Having heart disease	0.18 (−0.33, 0.68)	0.96 (0.74, 1.24)	1.02 (0.67, 1.55)	1.03 (0.73, 1.46)
Knowing someone with heart disease	0.54 (0.24, 0.85)	1.02 (0.87, 1.21)	0.90 (0.67, 1.21)	1.08 (0.90, 1.31)
Having high blood pressure	0.03 (−0.31, 0.37)	1.05 (0.87, 1.26)	0.90 (0.65, 1.25)	0.86 (0.69, 1.08)
Never smoked	Reference			
Former smoker	0.48 (0.14, 0.82)	1.08 (0.91, 1.29)	0.91 (0.65, 1.29)	1.12 (0.90, 1.39)
Current smoker	0.10 (−0.34, 0.54)	1.01 (0.80, 1.28)	1.14 (0.75, 1.74)	1.20 (0.93, 1.54)
Knowledge score (1 unit increase)	–	1.06 (1.01, 1.11)	1.17 (1.07, 1.26)	1.19 (1.13, 1.26)

a *Because of only 1 observation, the category “diverse” was not included in the analysis, n = 632*.

b *Higher score indicating higher knowledge, possible range: 0-11, minimum: 2, maximum: 11*.

c *Refers to question: What would you do when a close female person tells you during a phone call about having abdominal pain since an hour/sudden chest pain?*

d *Refers to question: Do you know how to perform a cardiopulmonary resuscitation? The answer option: “Yes, I can perform it myself or instruct someone else to perform it” was considered “ability to perform resuscitation.”*

*PR, prevalence ratio; CI, confidence interval; CPR, cardiopulmonary resuscitation*.

For women, the knowledge score was 0.75 points higher than for men. Age, educational level, having heart disease, and hypertension were not associated with knowledge score. The knowledge score for participants knowing someone with heart disease was about half a point higher when compared to people not knowing anyone with heart disease. The knowledge score of former smokers was almost half a point higher when compared to people who never smoked. There was no consistent difference in the knowledge score among the federal states (Table 3). Regarding the symptom chest pain in the phone call scenario, estimates for all variables except knowledge score indicated no association (Table 3).

Regarding the symptom abdominal pain there was no association with gender, having heart disease, knowing someone with heart disease, hypertension, and smoking. PR for convincing the women to call an ambulance increased with older age. When compared to people with the highest educational level, PR for convincing the women to call an ambulance was higher in people still studying/having no degree, but CI included the null effect. PR for convincing the women to call an ambulance was lower in Baden-Wuerttemberg compared to Saxony-Anhalt, but again CI included the null effect. Higher knowledge score was associated with higher PR for convincing the women to call an ambulance (Table 3). Regarding the ability to perform CPR, there was no association with having and knowing someone with heart disease, and hypertension. Women were less likely to perform CPR compared to men. PR for the ability to perform CPR decreased with older age. PR of stating to be able to perform CPR was lower for people with vocational training and still studying/having no degree when compared to people with the highest educational level. When compared to people who never smoked, PR was higher in former and current smokers. However, CI included the null effect. Higher knowledge score was associated with higher PR to be able to perform CPR. There was no consistent difference among the federal states (Table 3).

## Discussion

In our study, we found that participants had a good understanding of how to react in case of an AMI since the vast majority would call an ambulance. In contrast, when confronted with the description of a phone call in which somebody displayed symptoms of an AMI, the proportion of participants who would convince the women to call an ambulance was considerably lower. Partly, this could be explained by the order of the questions, as the phone-call-scenario was the first question in the survey. Higher knowledge score was associated with higher PR of an adequate reaction in multiple regression. However, knowledge might not translate directly into reacting appropriately when witnessing an AMI.

In our scenario, it is plausible that more participants chose to convince the women to call an ambulance when confronted with chest pain compared to abdominal pain since chest pain was more frequently recognized as AMI symptom than abdominal pain. This underlines the importance to educate about atypical symptoms of AMI. In fact, especially women present more often with only non-chest pain discomfort and the elderly experience more atypical symptoms (22–24).

### Knowledge Score

Exploring the knowledge about symptoms of AMI, we found a mean knowledge score of 7.3/11. Hence, the mean proportion of symptoms that were correctly identified as belonging to AMI was 66.4%. This is considerably higher than in a previous study conducted in Germany in 2006 that found a proportion of 45.7% of symptoms to be correctly identified (10). The reason for the observed difference could be that selection bias in our sample might have influenced the results as well as the limitation to four regions of Germany, while the former study used a representative quota method across Germany. Possibly, in the time span from 2006 to 2020 the German population might have increased their knowledge due to efforts to better inform the public and educational campaigns as organized by the “Deutsche Herzstiftung e. V.” (25).

Compared to the weighted mean of a sample of eight European countries, Russia, and Singapore, the proportion that we found in Germany is higher too (66.4 vs. 42.1%) (11).

When looking at the proportion of participants who identified a specific symptom correctly, the least well-known symptom “headache” was still known by 33.8%, which is higher than in the previously mentioned studies (11, 24).

The before mentioned study from Germany observed a lower knowledge of abdominal pain (10). One possible explanation is that our observed higher knowledge of abdominal pain was influenced by our study set up, since half of the participants had already read about the woman on the phone with abdominal pain when we evaluated the knowledge of the AMI symptoms. Indeed, about 61% of the participants who were assigned to the scenario with abdominal pain identified it as symptom, while only 43% of the participants who were assigned to the scenario with chest pain did so.

The trap symptom “sudden visual disturbances” was falsely attributed to AMI by 44.5% of participants. This can be compared to the population in two studies from the United States (31.9%, 58%) and one from South Korea (33,8%), with a weighted mean proportion of 32.2% (11, 15, 17, 26). In a sample of AMI patients in Germany from 2016, the proportion of misattribution was only 19.2% (27). While this might suggest that AMI patients are better informed about this symptom, different forms of recruiting and the different areas in Germany might also influence the results. The false attribution of the symptom to AMI in our sample might point to a confusion about this symptom specifically or be influenced by the confounder that the participants were generally inclined to attribute the listed symptoms to AMI, a phenomenon that has been described by Greenlund et al. (28). However, this phenomenon could also be present in the other studies that were listed before.

### Predictors of Knowledge of Symptoms of AMI

When analyzing predicting factors of knowledge of AMI symptoms, we found that factors that are in connection with a general interest in health are also predictors for a better knowledge of AMI symptoms:

(1) Being female was associated with higher knowledge similarly to the findings of previous studies (10, 14–16, 29).
(2) Higher knowledge in people who know someone with heart disease might be influenced by their increased interest and direct talks about the disease. This aligns with a study that found a better knowledge of at least one to four symptoms when relatives, acquaintances, or neighbors had a history of AMI (17).
(3) Being an ex-smoker in comparison to people who never smoked was positively associated with a higher knowledge scale. This might be a result of increased interest in healthy living. When comparing smokers to non-smokers we did not observe an effect.

While past literature observed an association for history of heart disease and a good knowledge of the symptoms, as well as for having coronary heart disease and a recommended heart attack knowledge, we did not observe an effect for having heart disease (15, 30). It is plausible that people with heart disease have a higher interest in AMI so it is not clear to us why this association could not be found in our survey. One possible reason could be that in light of the low response rate to our survey, the interest of the participants was generally higher than in the general population and therefore, the interest among participants with heart disease was similar to the interest of the other participants. However, since we could find an association for other factors related to interest, this might not be a sufficient explanation.

Similarly, we did not find an association between knowledge and hypertension which is a risk factor for AMI. This aligns with a previous study that did not find a significant association between hypertension and excellent knowledge (17).

While we found no association with age, previous studies found young or middle-aged adults to be most knowledgeable (14–17), except for one study that found people aged 14–35 years to be least knowledgeable which might be influenced by the inclusion of teenagers in the sample (10). Regarding the elderly in our study, the results might have been skewed by a selection bias since the observed participants were all mentally fit enough and resourceful enough to use the Internet in order to answer the online survey and additionally, there were only few people aged 70 or older in our sample.

We found that participants with the highest education had the highest knowledge score but the associations were very small including the null effect. Past literature observed a better knowledge of the AMI symptoms in adults with higher education (10, 14–17, 31).

We did not observe differences among the federal states. Our results indicate that differences among Federal States with high and low AMI mortality rates in Germany might not be explained by differences in knowledge of AMI symptoms.

### Predictors of Reaction to Symptoms of AMI

When the participants were confronted with the symptom sudden chest pain in the phone call scenario, we observed an association with higher knowledge score and convincing the woman to call an ambulance. All other variables were not associated with the outcome.

When confronted with the symptom abdominal pain, older age and higher knowledge score were associated with convincing the woman to call an ambulance. Older people might be more aware that abdominal pain could be related to a severe disease than younger people and hence be more likely to convince the woman to call an ambulance. People in the federal states with low AMI mortality were less likely to convince the woman to call an ambulance when compared to people in Saxony-Anhalt, the state with the highest AMI mortality. Even though this result included the null effect, which might be partly due to small sample size, it is highly interesting, since it was hypothesized that people in federal states with a higher mortality of AMIs would rather not call an ambulance. This should be explored in further studies.

Since our chosen approach of analyzing the reaction to AMI symptoms by describing a real-life situation has, to our knowledge, not been conducted in the same way in previous literature, it needs more research to better understand the results and the associated factors for our observation.

### Predictors of Ability to Perform CPR

Factors associated with performing CPR were a better knowledge of the symptoms, being male, and younger age. People having no degree/still in training or studying were less likely to perform CPR in comparison with people holding Master, diploma, or doctorate degree.

In Germany, training in CPR is mandatory when taking classes for a driving license and in most social and health-related professions. However, in contrast to many other countries, in Germany the population is not regularly trained in CPR (32). Trying to explain our predictors, it might be plausible that for younger people the time span since obtaining their driving license was smaller, so they were able to remember their training in CPR better and therefore showed an increased confidence.

Our results indicate that differences among federal states with high and low AMI mortality rates in Germany might not be explained by differences in the ability to perform CPR.

### Limitations and Strength

One limitation of our study is the online assessment, which might exclude mainly the elderly, who are not familiar with the internet. Additionally, because of the low response rate and contacting only non-responders from a former survey, selection bias must be assumed. A strength of the study is that we assessed the knowledge and first responder reaction not only by testing the participants directly about their knowledge/preferred reaction without a practical context but also with real-life scenarios. The description of everyday scenarios could provide a higher external validity since it is closer to the actual experience of a bystander. Furthermore, this study is the first survey to assess knowledge, reaction to AMI symptoms, and ability to perform CPR in German federal states with different AMI mortality rates.

## Conclusions

This study indicates rather no differences regarding knowledge and reaction to AMI symptoms as well as ability to perform CPR among different regions with high and low AMI mortality rates when taking into account sociodemographic as well as health and health-related factors. Only few of the other examined factors showed an association with the outcomes. Further studies should explore which factors could influence knowledge, reaction to symptoms, and ability to perform CPR. This study highlights that less known AMI symptoms should be included in health campaigns. Educating the public about AMI symptoms and CPR may not be sufficient for enabling bystanders to gauge a real-life situation correctly and act accordingly. Educational health campaigns should focus on conveying the information in a format that is close to a real-life situation in order to have most impact (9). Interventions for enhancing ability to perform CPR should be compulsory in regular intervals.

## Data Availability Statement

The raw data supporting the conclusions of this article will be made available by the authors, without undue reservation.

## Ethics Statement

This study was approved by the Ethics Committee of the Medical Faculty of the Martin-Luther University Halle-Wittenberg.

## Author Contributions

RM and SH developed the general idea of the study and prepared the study protocol. SH, RM, and BB developed the questionnaire. BB drafted the initial manuscript. NK conducted the statistical analyses and contributed sections on methods and results. All authors contributed to the interpretation of the findings. All authors revised and commented on the manuscript and read and approved the final version.

## Funding

This study was funded by the Ministry of Labor, Social Affairs, and Integration of the Federal State of Saxony-Anhalt (Chapter 0513 Title 685 76) and by internal resources of the Martin-Luther University Halle-Wittenberg.

## Conflict of Interest

The authors declare that the research was conducted in the absence of any commercial or financial relationships that could be construed as a potential conflict of interest.

## Publisher's Note

All claims expressed in this article are solely those of the authors and do not necessarily represent those of their affiliated organizations, or those of the publisher, the editors and the reviewers. Any product that may be evaluated in this article, or claim that may be made by its manufacturer, is not guaranteed or endorsed by the publisher.

## Abbreviations

IHD: ischemic heart disease
AMI: acute myocardial infarction
CPR: cardiopulmonary resuscitation
PR: prevalence ratio
CI: confidence interval.
