# Supplementary figures and images for: Knowledge of Symptoms of Acute Myocardial Infarction, Reaction to the Symptoms, and Ability to Perform Cardiopulmonary Resuscitation: Results From a Cross-sectional Survey in Four Regions in Germany
Source: Front Cardiovasc Med. 2022 May 16;9:897263. doi: 10.3389/fcvm.2022.897263 (PMC9148950; doi:10.3389/fcvm.2022.897263)

## Baden–Wuerttemberg

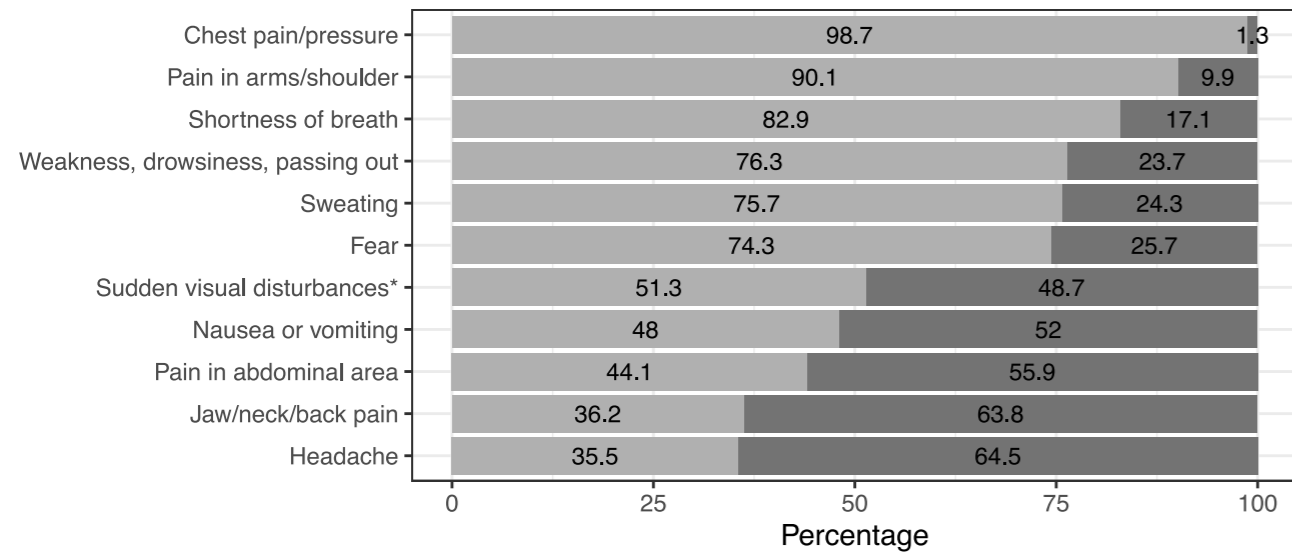

## North–Rhine Westphalia

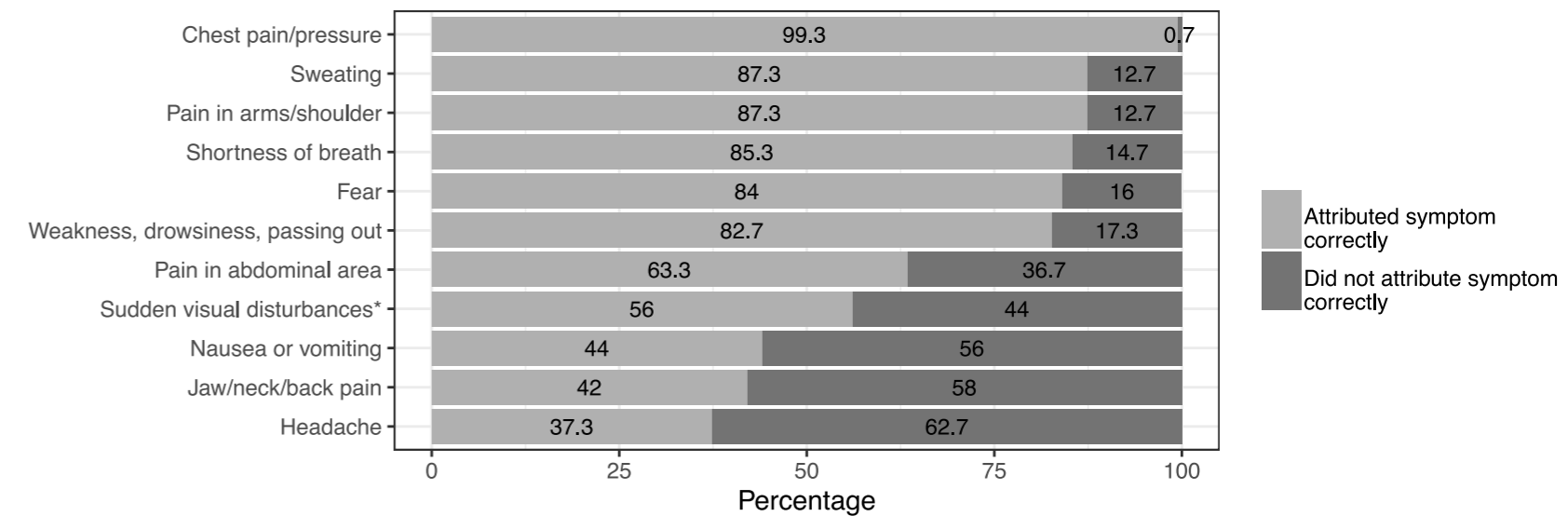

## Saxony–Anhalt

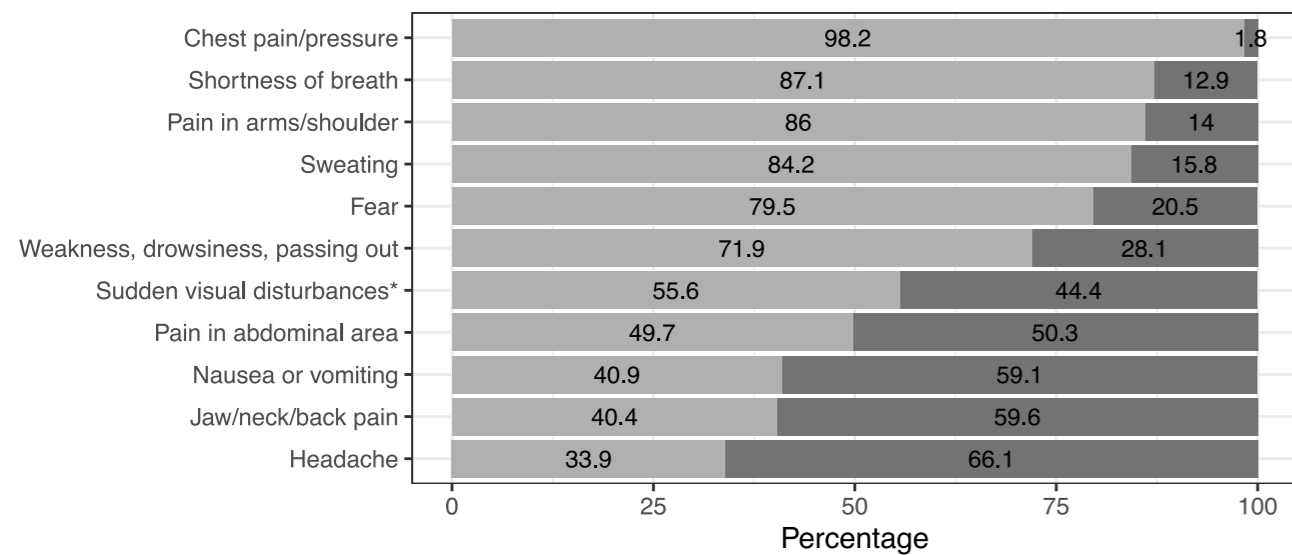

## Schleswig–Holstein

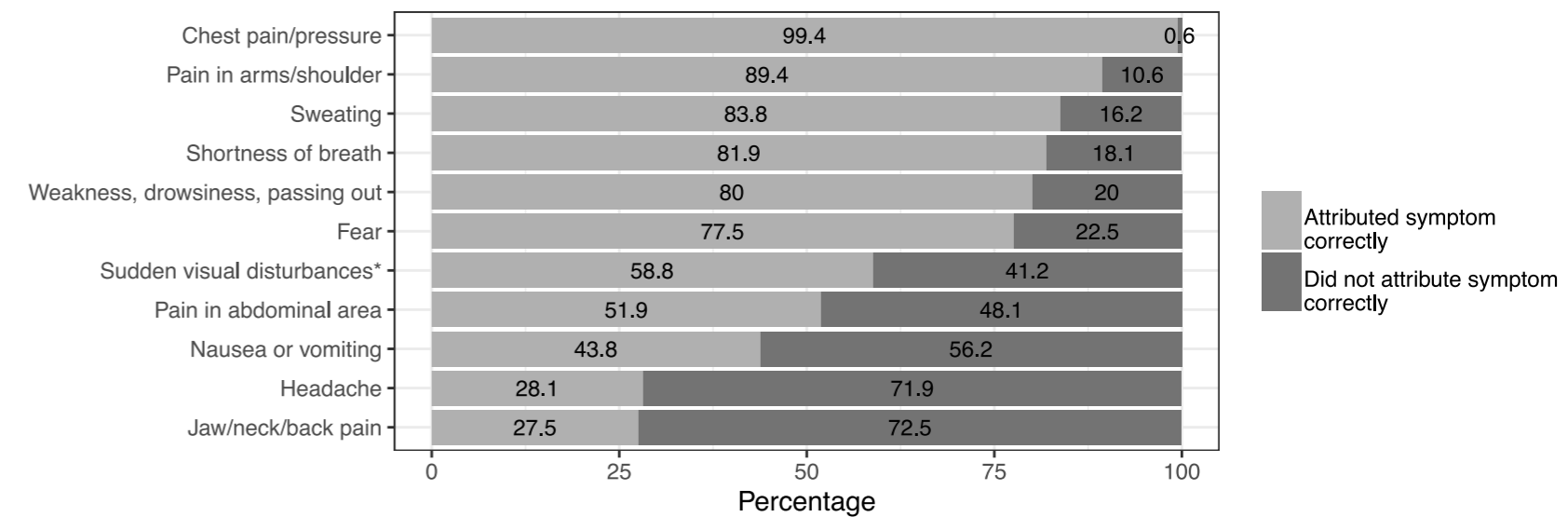

Supplement: Supplementary file 1 [file Data_Sheet_1.PDF]

### Baden–Wuerttemberg

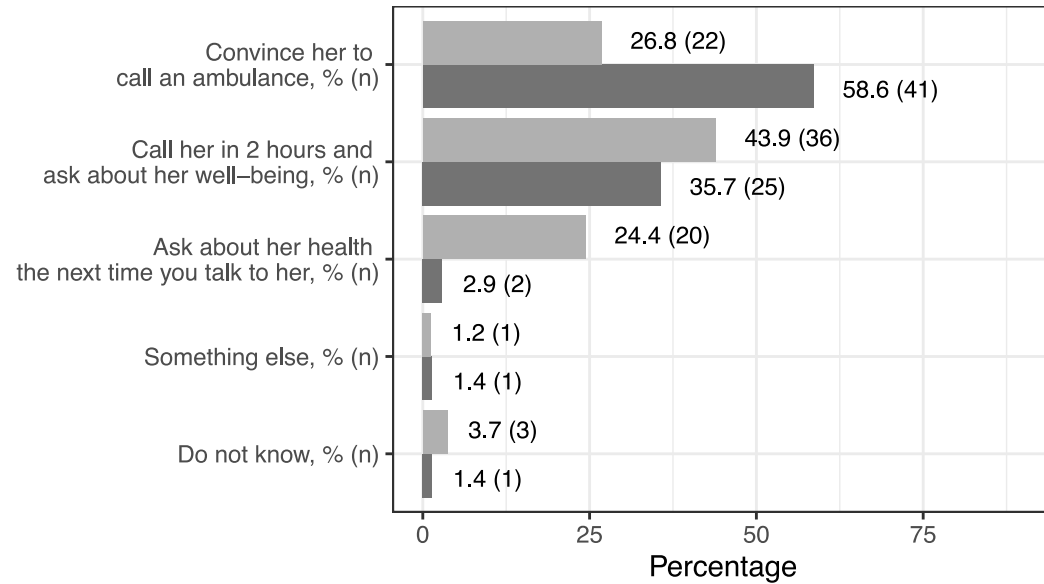

### North–Rhine Westphalia

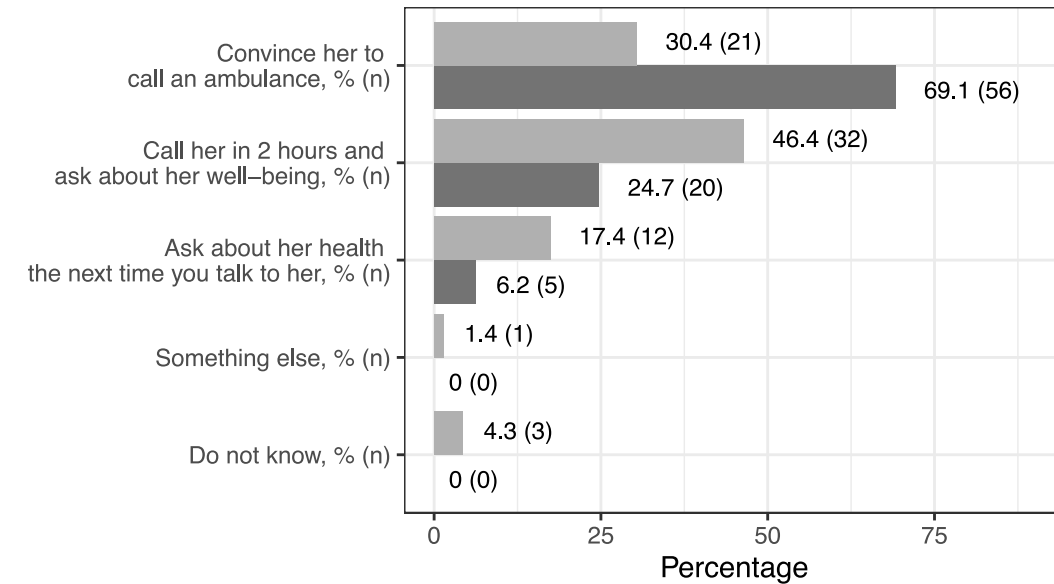

### Saxony–Anhalt

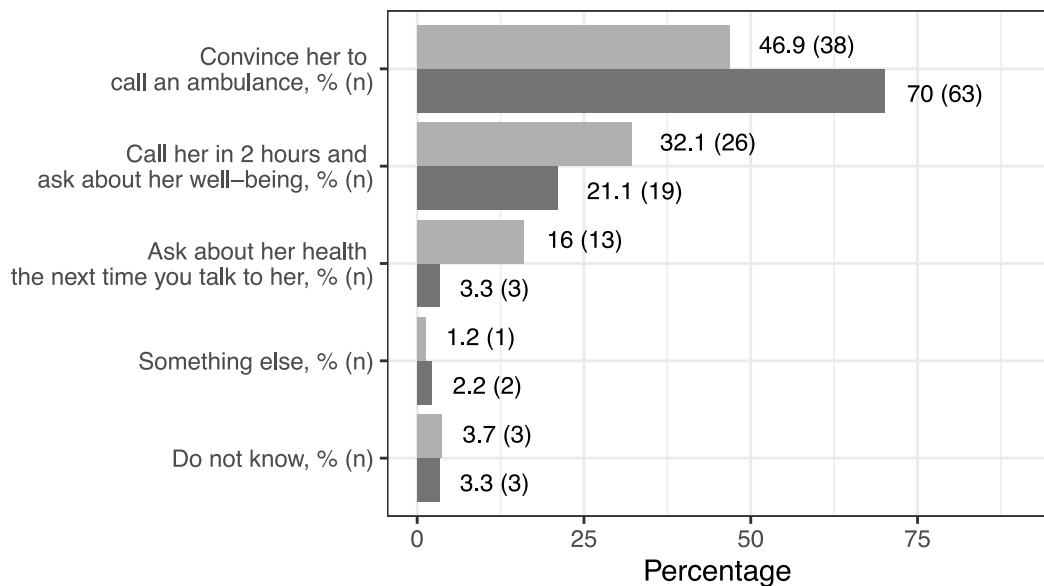

### Schleswig–Holstein

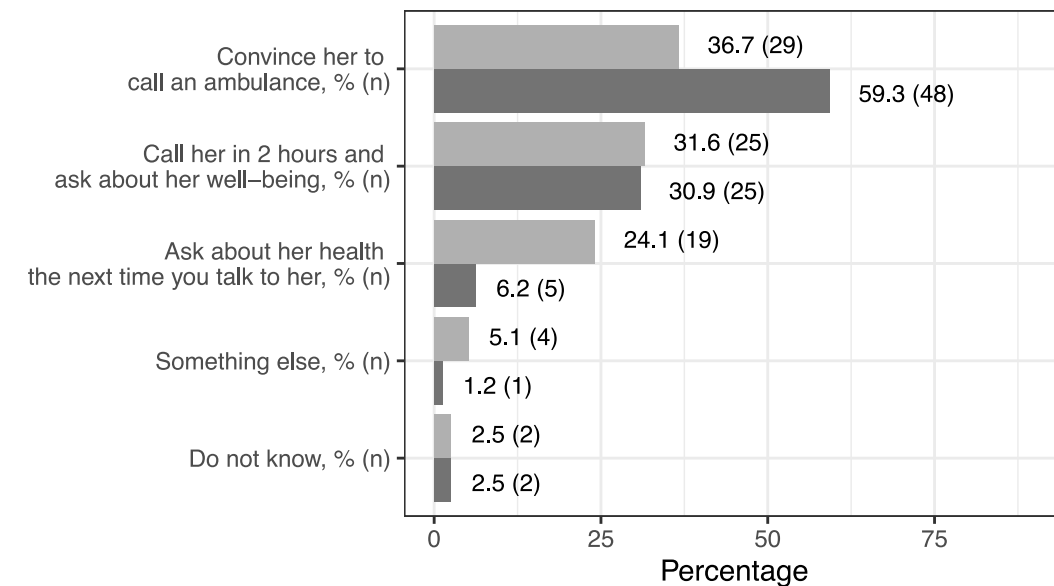

Supplement: Supplementary file 2 [file Data_Sheet_2.PDF]

Baden–Wuerttemberg

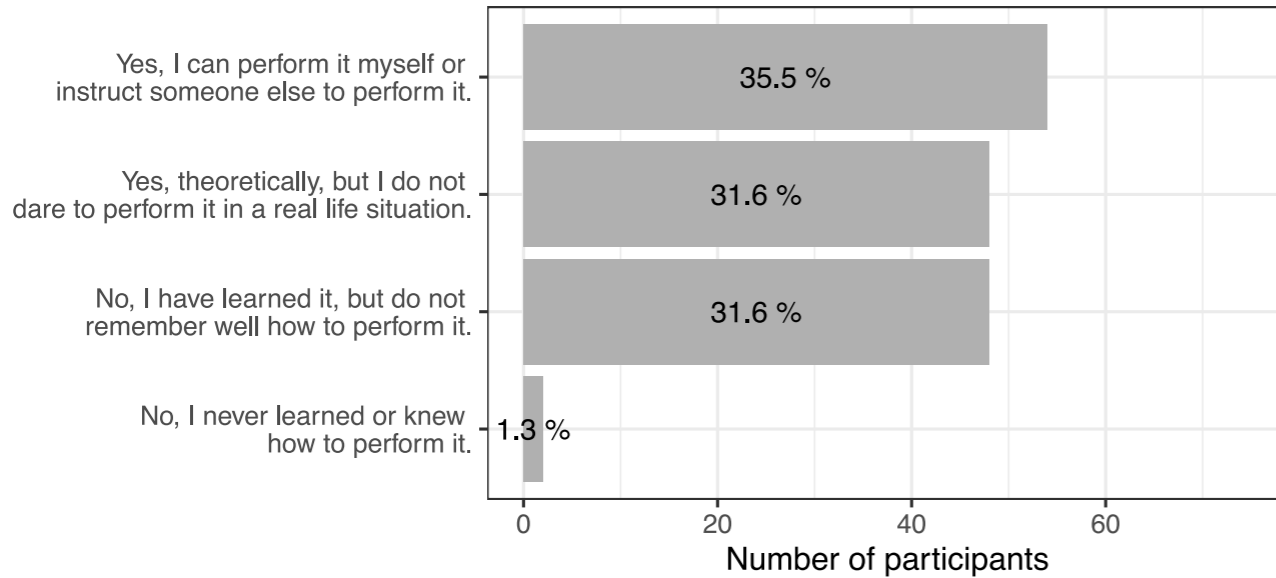

North–Rhine Westphalia

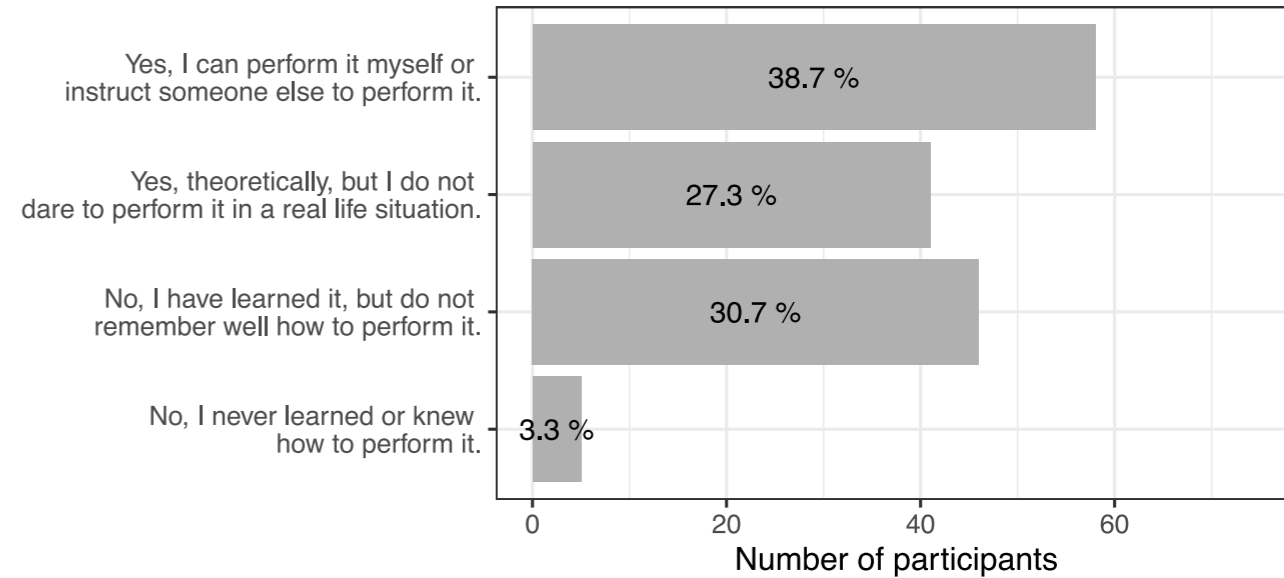

Saxony–Anhalt

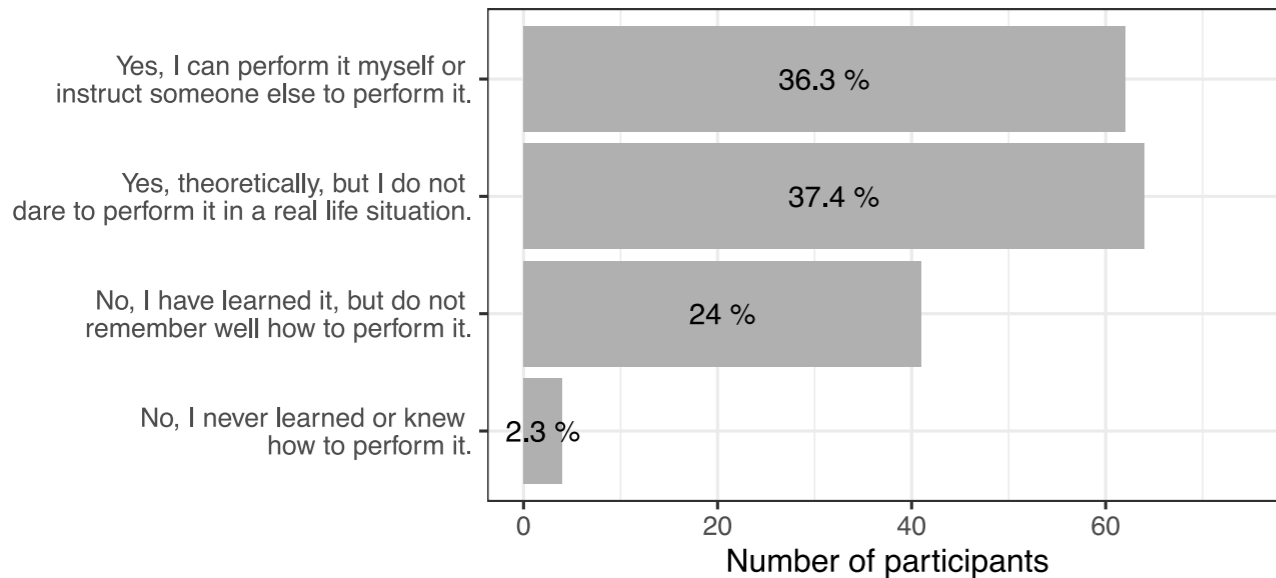

Schleswig–Holstein

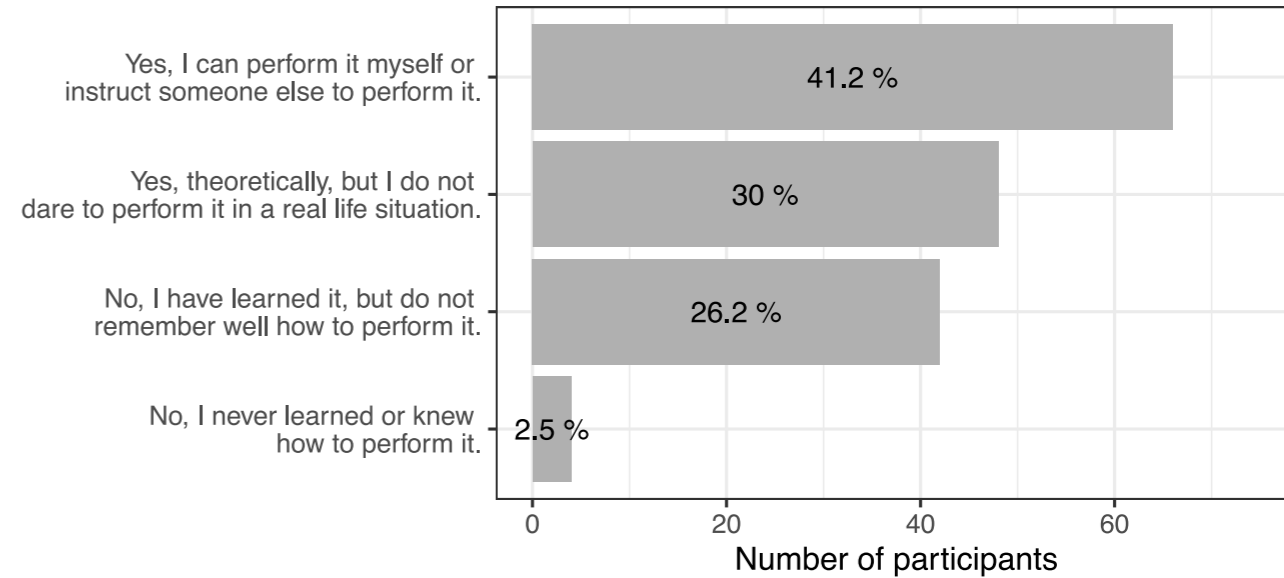

Supplement: Supplementary file 3 [file Data_Sheet_3.PDF]
