# Supplementary material for: Knowledge of Symptoms of Acute Myocardial Infarction, Reaction to the Symptoms, and Ability to Perform Cardiopulmonary Resuscitation: Results From a Cross-sectional Survey in Four Regions in Germany
Source: Front Cardiovasc Med. 2022 May 16;9:897263. doi: 10.3389/fcvm.2022.897263 (PMC9148950; doi:10.3389/fcvm.2022.897263)
Supplement: Supplementary file 4 [file Data_Sheet_4.PDF]

Additional File 4

Supplemental Table 1. Reaction to other people's symptoms of myocardial infarction (n = 633)

**Imagine you are in a department store and notice a person lying on the floor. Nobody else seems to have noticed the person.**	
I am not capable of gauging the situation correctly or to act by myself. Therefore, I ask for help, hoping someone else knows what to do, n (%)	39 (6.2)
I know the number of the ambulance service and call them, so they can tell me what to do, n (%)	67 (10.6)
I am capable of gauging the situation correctly and, if necessary, start the resuscitation, as I have learned it, n (%)	240 (37.9)
I check whether the person is responsive or breathing. If this is not the case, I call the ambulance. I do not dare starting resuscitation in case the person is not breathing, n (%)	271 (42.8)
Something else, n (%)	15 (2.4)
Do not know, n (%)	1 (0.2)
**When you think that someone has a cardiac infarction, what would be the first thing you would do?**	
Call the ambulance, n (%)	596 (94.2)
Bring the person to the hospital, n (%)	13 (2.1)
Recommend the person to consult a physician, n (%)	6 (0.9)
Contact a family member, n (%)	5 (0.8)
Something else, n (%)	13 (2.1)
