# Supplementary material for: Knowledge of Symptoms of Acute Myocardial Infarction, Reaction to the Symptoms, and Ability to Perform Cardiopulmonary Resuscitation: Results From a Cross-sectional Survey in Four Regions in Germany
Source: Front Cardiovasc Med. 2022 May 16;9:897263. doi: 10.3389/fcvm.2022.897263 (PMC9148950; doi:10.3389/fcvm.2022.897263)
Supplement: Supplementary file 5 [file Data_Sheet_5.PDF]

Additional File 5

Supplemental Table 2. Univariable regression for knowledge and reaction to symptoms of myocardial infarction and possible predictors

	Knowledge score, ß (95% CI)[b]	Would convince her to call ambulance in case of sudden chest pain (n = 322), PR (95% CI)[c]	Would convince her to call ambulance, in case of abdominal pain (n = 311), PR (95% CI)[c]	Ability to perform CPR, PR (95% CI)[d]
Male[a]	Reference			
Female[a]	0.79 (0.49, 1.09)	0.97 (0.83, 1.14)	1.04 (0.77, 1.40)	0.90 (0.74, 1.10)
Age (per 10 years)	-0.07 (-0.17, 0.03)	1.03 (0.98, 1.08)	1.09 (0.99, 1.21)	0.91 (0.86, 0.97)
Master, diploma, doctorate (PhD)	Reference			
Bachelor or equivalent	-0.11 (-0.53, 0.31)	0.98 (0.77, 1.24)	1.37 (0.92, 2.04)	1.06 (0.82, 1.37)
Vocational training	-0.10 (-0.46, 0.26)	1.07 (0.89, 1.29)	1.04 (0.71, 1.55)	0.91 (0.72, 1.15)
No degree/still in training or studying	0.09 (-0.60, 0.79)	0.97 (0.65, 1.44)	1.80 (1.09, 2.97)	0.70 (0.40, 1.22)
Saxony-Anhalt	Reference			
Baden-Wuerttemberg	-0.14 (-0.57, 0.28)	0.84 (0.66, 1.06)	0.57 (0.37, 0.88)	0.98 (0.73, 1.31)
North Rhine-Westphalia	0.41 (-0.02, 0.84)	0.99 (0.81, 1.20)	0.65 (0.42, 0.99)	1.07 (0.80, 1.42)
Schleswig-Holstein	-0.06 (-0.48, 0.37)	0.85 (0.68, 1.06)	0.78 (0.54, 1.13)	1.14 (0.87, 1.49)
Having heart disease	0.04 (-0.46, 0.55)	1.05 (0.82, 1.35)	1.17 (0.75, 1.83)	0.91 (0.64, 1.29)
Knowing someone with heart disease	0.67 (0.36, 0.97)	1.09 (0.92, 1.28)	0.97 (0.72, 1.32)	1.13 (0.92, 1.38)
Having high blood pressure	-0.12 (-0.44, 0.20)	1.04 (0.88, 1.22)	1.08 (0.79, 1.48)	0.79 (0.63, 0.99)
Never smoked	Reference			
Former smoker	0.37 (0.03, 0.71)	1.12 (0.94, 1.33)	1.07 (0.76, 1.50)	1.13 (0.90, 1.41)
Current smoker	-0.10 (-0.54, 0.39)	1.02 (0.80, 1.30)	1.21 (0.80, 1.82)	1.24 (0.94, 1.62)
Knowledge score (1 unit increase)	-	1.05 (1.01, 1.09)	1.17 (1.08, 1.27)	1.20 (1.14, 1.27)

[a]Because of only 1 observation, the category "diverse" was not included in the analysis, n = 632
[b]Higher score indicating higher knowledge, possible range: 0-11, minimum: 2, maximum: 11
[c]Refers to question: What would you do when a close female person tells you during a phone call about having abdominal pain since an hour/sudden chest pain?
[d]Refers to question: Do you know how to perform a reanimation? The answer option: “Yes, I can perform it myself or instruct someone else to perform it” was considered “ability to perform reanimation”; PR, prevalence ratio; CI, confidence interval; CPR, cardiopulmonary resuscitation
